# Time-course effects of aerobic exercise training on cardiovascular and renal parameters in 2K1C renovascular hypertensive rats

**DOI:** 10.1590/1414-431X20154499

**Published:** 2015-08-11

**Authors:** R.C.A. Maia, L.E. Sousa, R.A.S. Santos, M.E. Silva, W.G. Lima, M.J. Campagnole-Santos, A.C. Alzamora

**Affiliations:** 1 Departamento de Ciências Biológicas, Instituto de Ciências Exatas e Biológicas, Universidade Federal de Ouro Preto, Ouro Preto, MG, Brasil; 2 Programa de Pós-Graduação em Saúde e Nutrição, Universidade Federal de Ouro Preto, Ouro Preto, MG, Brasil; 3 Programa de Pós-Graduação em Ciências Biológicas, NUPEB, Universidade Federal de Ouro Preto, Ouro Preto, MG, Brazil; 4 Departamento de Fisiologia e Biofísica, Instituto de Ciências Biológicas, Universidade Federal de Minas Gerais, Belo Horizonte, MG, Brasil

**Keywords:** 2K1C renovascular hypertension, Swimming, Baroreflex bradycardia, Heart and kidney adaptations, Oxidative stress

## Abstract

Exercise training (Ex) has been recommended for its beneficial effects in hypertensive states. The present study evaluated the time-course effects of Ex without workload on mean arterial pressure (MAP), reflex bradycardia, cardiac and renal histology, and oxidative stress in two-kidney, one-clip (2K1C) hypertensive rats. Male Fischer rats (10 weeks old; 150–180 g) underwent surgery (2K1C or SHAM) and were subsequently divided into a sedentary (SED) group and Ex group (swimming 1 h/day, 5 days/week for 2, 4, 6, 8, or 10 weeks). Until week 4, Ex decreased MAP, increased reflex bradycardia, prevented concentric hypertrophy, reduced collagen deposition in the myocardium and kidneys, decreased the level of thiobarbituric acid-reactive substances (TBARS) in the left ventricle, and increased the catalase (CAT) activity in the left ventricle and both kidneys. From week 6 to week 10, however, MAP and reflex bradycardia in 2K1C Ex rats became similar to those in 2K1C SED rats. Ex effectively reduced heart rate and prevented collagen deposition in the heart and both kidneys up to week 10, and restored the level of TBARS in the left ventricle and clipped kidney and the CAT activity in both kidneys until week 8. Ex without workload for 10 weeks in 2K1C rats provided distinct beneficial effects. The early effects of Ex on cardiovascular function included reversing MAP and reflex bradycardia. The later effects of Ex included preventing structural alterations in the heart and kidney by decreasing oxidative stress and reducing injuries in these organs during hypertension.

## Introduction

Structural and functional alterations in the heart and kidney are involved in the development of arterial hypertension by hyperactivity of the sympathetic nervous system and renin-angiotensin system (RAS) as well as their contributions to high blood pressure and reduced sensitivity of the baroreflex control of the heart rate (HR) (1,2). Many studies (1–4) have used the two-kidney, one-clip (2K1C) Goldblatt hypertensive model in an attempt to understand the mechanisms of development and maintenance of renovascular hypertension. The time course of the 2K1C hypertensive model has been divided into several phases after clipping of the renal artery: at about 4 weeks, blood pressure rises in association with increases in the plasma renin activity and circulating angiotensin II (Ang II) concentration. In weeks 5 to 8, hypertension is associated with increases in tissue RAS components despite a fall in plasma renin activity and circulating Ang II. At week 9 and after, hypertension is maintained by increases in the tissue RAS activity, plasma volume, and sympathetic tone (1). Moreover, evidence has shown increased generation of reactive oxygen species (ROS) in specific organs such as the brain, heart, and kidneys during renovascular hypertension (3,4).

Ang II, aldosterone, and catecholamines are involved in the development of ventricular hypertrophy under pathological (5,6) and physiological (7,8) conditions, reflected as worsening or improvement of cardiac function, respectively. Ventricular hypertrophy can be concentric in certain pathological conditions, such as arterial hypertension, or concentric and eccentric in physiological cardiac hypertrophy induced by static or dynamic physical exercise training (Ex), which induces two different types of intermittent chronic cardiac workload (6,8,9).

Ex induces adaptive cardiovascular benefits in hypertensive conditions by reducing the sympathetic outflow, vascular resistance, and plasma Ang II levels and improving the sensitivity of the baroreflex (2,10–13). Additionally, during endurance Ex, the increase in oxygen consumption results in increased generation of ROS, which is involved in the adaptive up-regulation of antioxidant gene expression (14). Moreover, evidence has shown that low-intensity Ex (50–60% of maximal exercise capacity) more effectively decreases blood pressure in hypertensive patients and rats than does high-intensity Ex (7,15–17). However, to maintain these benefits over time, close monitoring by healthcare professionals is required to adjust the Ex intensity to avoid possible adverse effects of more vigorous exercise, especially in hypertensive states, considering that risk factors such as age and cardiac disease could be associated with this pathology (18–20).

In the present study, our hypothesis was that Ex performed without adjusting the workload over time, even if it does not effectively reduce the blood pressure, could have beneficial effects on organs that participate in the control of blood pressure and thus reduce the cardiovascular risk. In view of these considerations, we evaluated the time-course effects of Ex without workload on the mean arterial pressure (MAP), reflex bradycardia, cardiac and renal histology, and oxidative stress at different stages of development of 2K1C hypertension.

## Material and Methods

### Ethics approval

All experiments were performed on 123 male Fischer rats (10 weeks of age; 150–180 g) from ENUT, Universidade Federal de Ouro Preto, MG, Brasil. The animals were housed in separate cages in groups of four (2K1C or SHAM) with free access to rat chow and tap water in a temperature- and light-controlled room (24±1°C; 12:12 h light-dark cycle). All animal procedures were in accordance with the Guidelines for Ethical Care of Experimental Animals and performed as approved by the Institutional Ethics Committee of the Universidade Federal de Ouro Preto (Protocol #022/2007).

### Induction of renovascular hypertension

Renovascular hypertension was induced as described by Goldblatt et al. (21). Briefly, the rats were anesthetized with a mixture of ketamine (50 mg/kg) and xylazine (10 mg/kg, *ip*), and a silver clip (inner diameter, 0.20 mm) was placed around the left renal artery through a midline incision (2K1C). The other rats were submitted to similar procedures but without the renal artery clip placement (SHAM group or normotensive rats).

### Physical Ex protocol

Four days after surgery (SHAM or 2K1C), the rats were subjected to swimming Ex without a workload for 2, 4, 6, 8 or 10 weeks for 1 h/day, 5 days/week. For adaptive purposes, the rats swam for 20 min on day 1, 40 min on day 2, and 1 h from day 3 until the end of training period. The Ex was performed in groups of four or five rats in a 38- × 60- × 50-cm tank. Water temperature was maintained at approximately 30±2°C, controlled by a thermostat. Sedentary (SED) rats were placed in the swimming apparatus with shallow water for 1 h/day, 5 days/week to mimic the water stress associated with the experimental protocol. The Ex protocol was performed according to a previously described method (22).

### Arterial pressure measurements

Forty-eight hours after the end of the Ex and SED protocols, the rats were anesthetized with urethane (1.2 g/kg body weight, *ip*; Sigma-Aldrich, USA). Next, a polyethylene catheter was inserted into the abdominal aorta through the femoral artery to measure the arterial pressure, and another catheter was inserted into the inferior vena cava through the femoral vein for injection of drugs to evaluate the baroreflex sensitivity (23). Anesthesia was intravenously supplemented thereafter. The adequate depth of anesthesia was determined by observing the corneal and paw pinch reflexes. Pulsatile arterial pressure was monitored by a Gould pressure transducer (PM-1000; CWE, USA) coupled to a blood pressure signal amplifier (UIM100A PowerLab System; ADInstruments, New Zealand). MAP and HR were determined from the arterial pressure wave. All variables were continuously recorded with a PowerLab digital acquisition system (Power Lab 4/20; ADInstruments) with an 800-Hz sampling rate.

### Evaluation of baroreflex bradycardia

The sensitivity of the baroreflex control of HR was determined by recording reflex HR changes in response to transient increases (baroreflex bradycardia) in MAP produced by repeated bolus injections of graded doses of phenylephrine (0.5–50.0 µg, *iv*). The HR was converted to the pulse interval (PI, ms) by the following formula: PI=60,000/HR. A best-fit regression line was drawn from the MAP and HR changes obtained with the different doses of phenylephrine for each animal. The slope of the regression line was used as an index of baroreflex sensitivity (baroreflex gain), as in previous studies (23).

### Analysis of cardiac and renal structures

For the histopathological analysis, hearts and kidneys were collected and fixed in 10% neutral-buffered formalin solution. After 72 h of fixation, the hearts and kidneys were dehydrated, cleared, and embedded in paraffin. The paraffin block was cut into 4- to 5-μm-thick sections, and adjacent sections were stained with either hematoxylin/eosin for evaluation of general myocardial and renal damage or Masson's trichrome for quantification of collagen-tissue deposition. Morphometric evaluations were made in tissue sections under an optical microscope (DM5000; Leica, Germany) and analyzed with QWin Image Processing and Analysis Software (Leica) in 20 optical microscope images at 40× magnification for each animal. In the hearts, the cardiomyocyte diameter was measured by a previously described method (2) in 20 optical microscope images at 40× magnification. The left ventricle wall thickness (Wt) and ventricle lumen (L) were measured on sections at 5× magnification, and the degree of cardiac hypertrophy was calculated as the Wt/L ratio. Higher Wt/L ratios indicated concentric hypertrophy, and lower Wt/L ratios indicated eccentric hypertrophy. Because the SHAM SED rats did not show changes in these ratios, the Wt/L ratio of these animals was used as a control. The cardiac and renal inflammatory process and tissue collagen deposition were also quantified as previously described (2).

### Analysis of oxidative damage

In the other groups of animals, the level of thiobarbituric acid-reactive substances (TBARS) and catalase (CAT) activity were analyzed at the end of 4 or 8 weeks of the Ex protocol. The left ventricle was perfused with 0.9% saline, and the heart and kidneys were collected and stored on crushed ice in labeled tubes. The organs were then homogenized in 1 mL of potassium phosphate buffer, pH 7.5, and centrifuged at 1500 *g* for 10 min. The supernatant was collected and the final volume of all samples adjusted to 1.5 mL with phosphate buffer. The samples were stored in a freezer for later biochemical analysis (24).

We used the formation of TBARS during an acid-heating reaction as an index of lipid peroxidation (25). Briefly, the samples from homogenates were mixed with 1 mL of 10% trichloroacetic acid and 1 mL of 0.67% thiobarbituric acid and then heated in a boiling water bath for 30 min. The TBARS level was determined by the absorbance at 532 nm and reported as malondialdehyde equivalents (U/mg protein).

The organ homogenates were used to determine the CAT activity, which was measured by the rate of decrease of H_2_O_2_ at 240 nm. The total protein content in the organ homogenate samples was determined using the Bradford method (26).

### Statistical analysis

The results are reported as means±SE. TBARS and catalysis data were analyzed using the Shapiro-Wilk normality test. Other data were analyzed for Kolmogorov-Smirnov normality and followed the standard normal distribution; they were subsequently assessed by two-way ANOVA followed by the Bonferroni post-test. Pearson correlation coefficients were used for correlation analysis. Statistical analyses were performed with the software GraphPad Prism (version 5.0; GraphPad Software, USA). The criterion for statistical significance was set at P<0.05.

## Results

### Baseline MAP and HR

**Figure 1:**
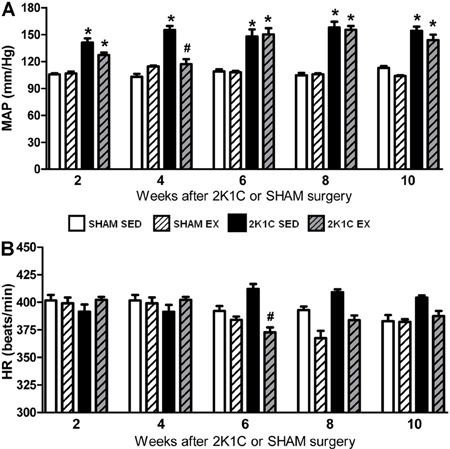
Baseline values of mean arterial pressure (MAP, *A*) and heart rate (HR, *B*) of anesthetized normotensive (SHAM, n=5−8) and hypertensive (2K1C, n=5−8) rats, either sedentary (SED) or subjected to physical exercise training (Ex), after 2, 4, 6, 8, and 10 weeks of swimming. *P<0.05 compared to SHAM SED rats. ^#^P<0.05 compared to 2K1C SED rats (two-way ANOVA followed by Bonferroni test).

The baseline MAP of all 2K1C SED rats was higher than the baseline MAP of all SHAM SED rats from week 2 to week 10 (P<0.05). A significant interaction between hypertension and Ex was observed in weeks 2 and 4 (P<0.05). The MAP in 2K1C Ex rats was significantly lower than that in 2K1C SED rats (P<0.05) and reached a level similar to that in the SHAM SED group only in week 4. In weeks 6, 8, and 10, the 2K1C Ex rats had a baseline MAP similar to that of the 2K1C SED rats (P>0.05) and higher than that of the SHAM SED rats (Figure 1A). In all weeks, there was no difference between the SHAM SED and SHAM Ex rats (P>0.05) (Figure 1A).

The baseline HR of SHAM Ex rats was similar to that of the SHAM SED rats in all weeks (P>0.05). In week 6, however, the baseline HR of the 2K1C Ex rats was significantly lower than that of the 2K1C SED rats (P<0.05) (Figure 1B). Furthermore, the HR was lower at 6, 8, and 10 weeks than at 2 weeks for both groups submitted to Ex (SHAM Ex and 2K1C Ex).

### Evaluation of baroreflex bradycardia

**Figure 2:**
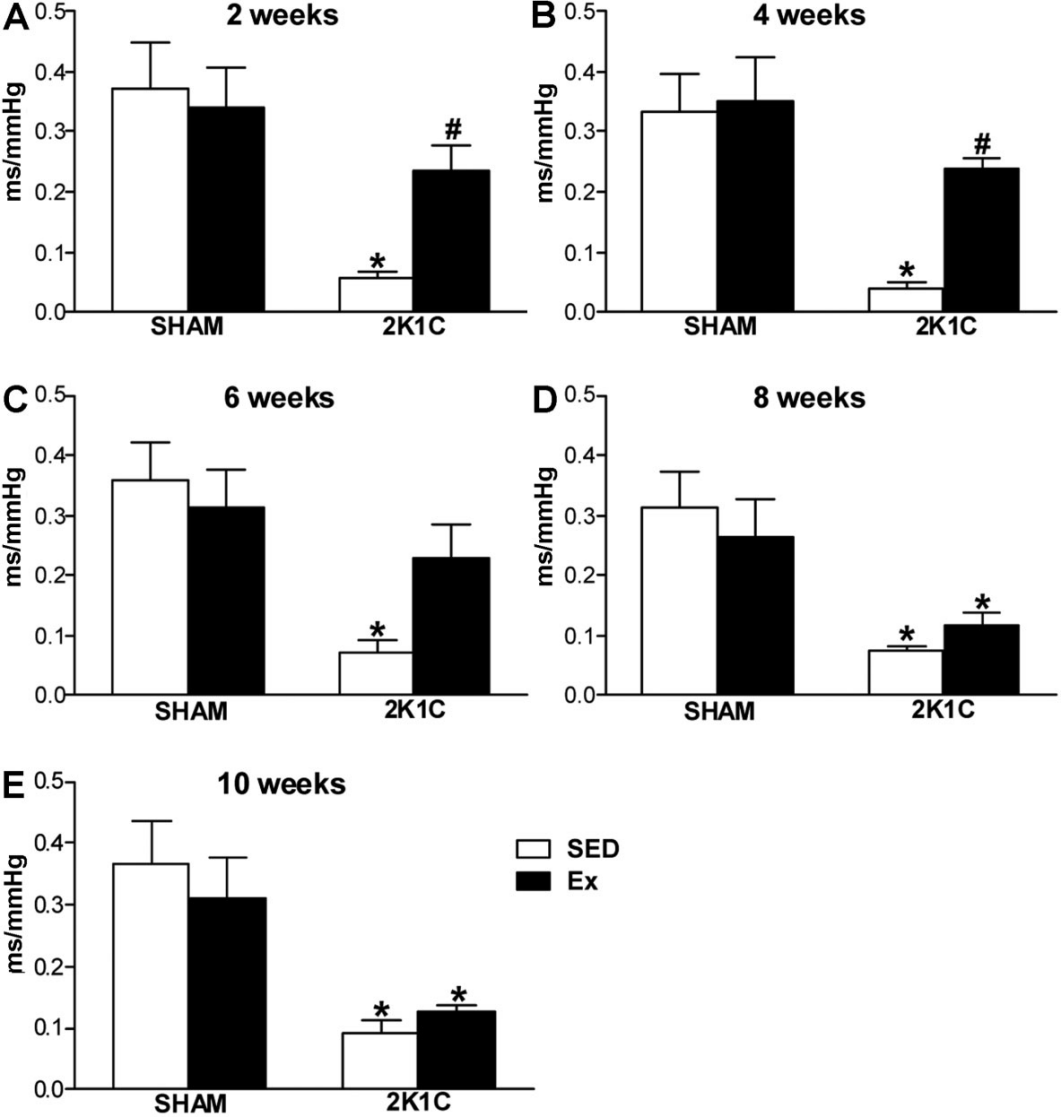
Index of the sensitivity of baroreflex bradycardia (ms/mmHg) induced by injection of phenylephrine (4.0 mg, *iv*) in normotensive (SHAM, n=5−8) and hypertensive rats (2K1C, n=5−8), either sedentary (SED) or subjected to physical exercise training (Ex), at 2 (*A*), 4 (*B*), 6 (*C*), 8 (*D*), and 10 weeks (*E*) of swimming. *P<0.05 compared to SHAM SED rats. ^#^P<0.05 compared to 2K1C SED rats (two-way ANOVA followed by Bonferroni test).

As expected, the sensitivity of reflex bradycardia in 2K1C SED rats was lower than that in SHAM SED rats in all weeks (P<0.05). However, the reflex bradycardia in the 2K1C Ex rats was higher than that of 2K1C SED rats (P<0.05) and similar to that of SHAM SED rats (P>0.05) at weeks 2 and 4 (Figure 2A and B). At week 6, the reflex bradycardia in 2K1C Ex rats was similar to that in 2K1C SED and SHAM SED rats (P>0.05) (Figure 2C). Furthermore, at weeks 8 and 10, Ex did not improve the reflex bradycardia in 2K1C rats compared with 2K1C SED rats (P>0.05) (Figure 2D and E). No change was observed in the sensitivity of reflex bradycardia between SHAM SED and SHAM Ex rats (P>0.05) (Figure 2A-D). Our data also showed that the baseline blood pressure correlated inversely (P<0.05) with the sensitivity of the reflex at weeks 2 (r=-0.9768), 4 (r=-0.9524), and 8 (r=-0.9806).

### Analysis of cardiac structure

**Figure 3:**
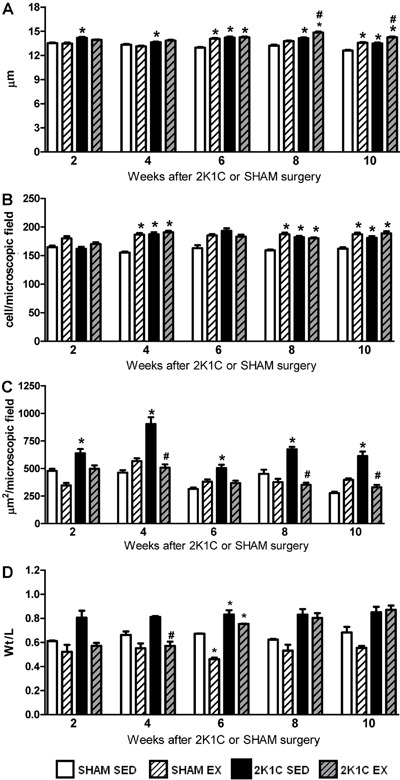
Cardiomyocyte diameter (µm, *A*), number of myocardial inflammatory cells per microscopic field (*B*), myocardial collagen deposition (µm^2^/microscopic field, *C*), and left ventricle wall thickness/lumen ratio (Wt/L, *D*) of normotensive (SHAM, n=3−7) and hypertensive (2K1C, n=3−5) rats, either sedentary (SED) or subjected to physical exercise training (Ex), for 2, 4, 6, 8, and 10 weeks of swimming. *P<0.05 compared to SHAM SED rats. ^#^P<0.05 compared to 2K1C SED rats (two-way ANOVA followed by Bonferroni test).

**Figure 4:**
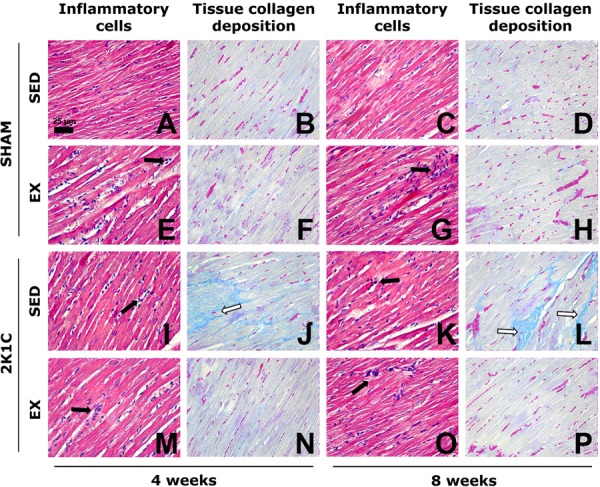
Photomicrographs of the myocardium of normotensive (SHAM) and hypertensive (2K1C) rats, either sedentary (SED) or subjected to physical exercise training (Ex, swimming) for 4 and 8 weeks. The number of inflammatory cells is consistent with the framework of normality in SHAM SED rats (*A* and *C*). The number of inflammatory cells (black arrows) is increased in SHAM Ex (*E* and *G*), 2K1C SED (*I* and *K*), and 2K1C Ex rats (*M* and *O*). The area with collagen deposition is compatible with normality in SHAM SED rats (*B* and *D*). Note the large area of collagen deposition (white arrows) in 2K1C rats (*J* and *L*) and small area of collagen deposition, similar to the normal pattern of SHAM SED rats, in 2K1C Ex rats (*N* and *P*). *A*, *E*, *I*, *M*, *C*, *G*, *K*, and *O* are stained with hematoxylin and eosin. *B*, *F*, *J*, *N*, *D*, *H*, *L*, and *P* are stained with Masson trichrome. All photomicrographs are magnified to 440× (bar=25 µm).

From week 2 to week 10, the wet relative heart weights of 2K1C SED rats (0.39±0.01 g/100 g body weight, n=27) were significantly higher than those of the SHAM SED rats (0.31±0.01 g/100 g body weight, n=30; P<0.05) and similar to those of the 2K1C Ex rats (0.39±0.02 g/100 g body weight, n=45; P>0.05). Also from week 2 to week 10, the cardiomyocyte diameter in the 2K1C SED rats was significantly greater than that of the SHAM SED rats (P<0.05). At week 6, the SHAM Ex and 2K1C Ex rats showed a significantly greater cardiomyocyte diameter than that of the SHAM SED rats (P<0.05). However, at weeks 8 and 10, the 2K1C Ex rats showed a significantly greater cardiomyocyte diameter than that of the SHAM SED and 2K1C SED rats (P<0.05) (Figure 3A). Additionally, at weeks 4, 8, and 10, the 2K1C SED, 2K1C Ex, and SHAM Ex rats showed higher numbers of myocardial inflammatory cells than did the SHAM SED rats (Figures 3B and 4).

The tissue deposition of collagen in the myocardium of 2K1C SED rats was significantly higher than that of SHAM SED rats from week 2 to week 10 (P<0.05). However, Ex prevented myocardial collagen deposition in 2K1C rats because these values remained significantly lower than those in the 2K1C SED rats (P<0.05) and similar to those in the SHAM SED rats (P>0.05) at weeks 4, 8, and 10 (Figures 3C and 4). At week 4, the Wt/L ratio of the left ventricle was significantly lower in 2K1C Ex rats than in 2K1C SED rats (P<0.05) and similar between 2K1C Ex and SHAM Ex rats (P>0.05). However, at weeks 6, 8, and 10, the Wt/L ratio of the left ventricle was similar between the 2K1C Ex and 2K1C SED rats (P>0.05). At week 6, the Wt/L ratio of the left ventricle was significantly lower in SHAM Ex rats than in SHAM SED rats (P<0.05), suggesting eccentric hypertrophy (Figure 3D).

### Analysis of renal structure

**Figure 5:**
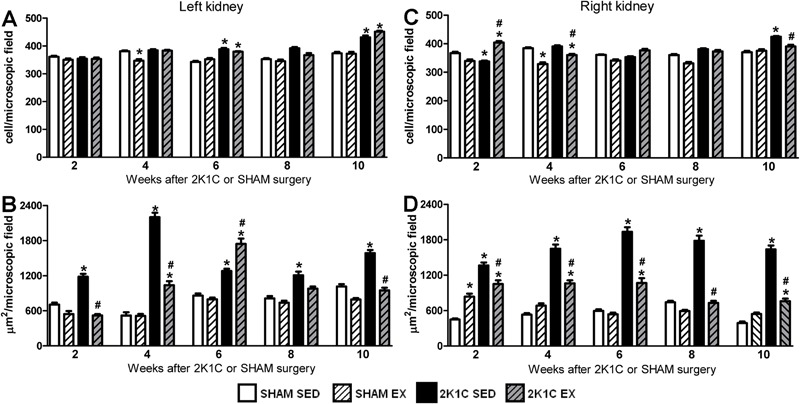
*A* and *C*, number of inflammatory cells per microscopic field in left (clipped) and right (non-clipped) kidneys, respectively. *B* and *D*, collagen deposition (µm^2^/microscopic field) in left and right kidneys, respectively, of normotensive (SHAM, n=3−6) and hypertensive (2K1C, n=3−5) rats, either sedentary (SED) or subjected to physical exercise training (Ex), for 2, 4, 6, 8, and 10 weeks of swimming. *P<0.05 compared to SHAM SED rats. ^#^P<0.05 compared to 2K1C SED rats (two-way ANOVA followed by Bonferroni test).

**Figure 6:**
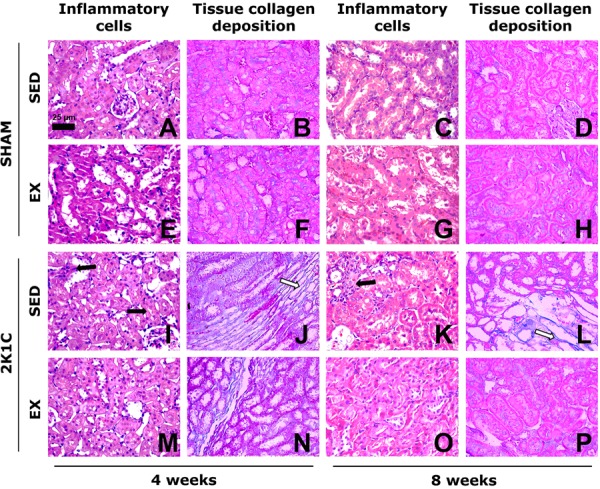
Photomicrographs of the left kidney of normotensive (SHAM) rats and hypertensive (2K1C) rats, either sedentary (SED) or subjected to physical exercise training (Ex, swimming) for 4 and 8 weeks. The number of inflammatory cells is consistent with the framework of normality in SHAM SED rats (*A* and *C*). The inflammatory process (black arrows) observed in the left kidney in SHAM Ex rats at week 8 (*G*), 2K1C SED rats (*I* and *K*), and 2K1C Ex rats (*M* and *O*) is similar to the normal pattern observed in SHAM SED rats. Note the area with collagen deposition compatible with normality in SHAM SED rats (*B* and *D*). Note the large area of collagen deposition (white arrows) in 2K1C rats (*J* and *L*) and small area of collagen deposition in 2K1C Ex rats at week 4 (*N*); however, at week 8, the area of collagen deposition in 2K1C Ex rats resembled the normal pattern of SHAM SED rats (*P*). *A*, *E*, *I*, *M*, *C*, *G*, *K*, and *O* are stained with hematoxylin and eosin. *B*, *F*, *J*, *N*, *D*, *H*, *L*, and *P* are stained with Masson trichrome. All photomicrographs are magnified to 440× (bar=25 µm).

**Figure 7:**
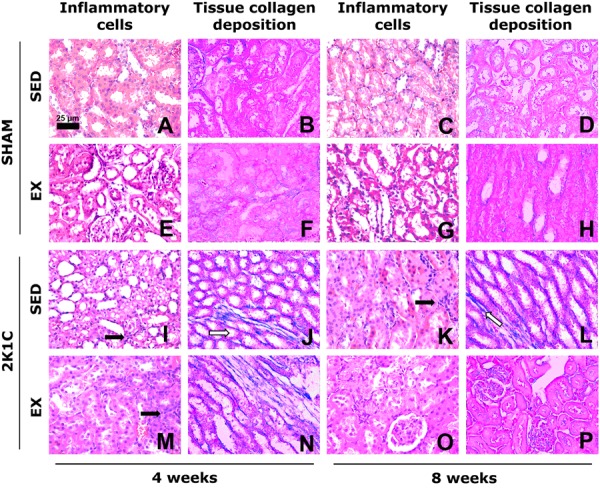
Photomicrographs of the right kidney of normotensive (SHAM) rats and hypertensive (2K1C) rats, either sedentary (SED) or subjected to physical exercise training (Ex, swimming) for 4 and 8 weeks. The number of inflammatory cells is consistent with the framework of normality in SHAM SED rats (*A* and *C*, respectively). The number of inflammatory cells decreased in SHAM Ex rats at week 4 (*E*) and in 2K1C Ex rats at week 4 (*M*). The inflammatory process (black arrows) observed in the right kidney in SHAM Ex rats at week 8 (*G*), 2K1C SED rats (*I* and *K*), and 2K1C Ex rats (*M* and *O*) was similar to the normal pattern observed in SHAM SED rats. Note the area of collagen deposition compatible with normality in SHAM SED rats (*B* and *D*). Note the large area of collagen deposition (white arrows) in 2K1C rats (*J* and *L*) and small area of collagen deposition in 2K1C Ex rats (*N* and *P*). *A*, *E*, *I*, *M*, *C*, *G*, *K*, and *O* are stained with hematoxylin and eosin. *B*, *F*, *J*, *N*, *D*, *H*, *L*, and *P* are stained with Masson trichrome. All photomicrographs are magnified to 440× (bar=25 µm).

From week 2 to week 10, the relative wet weight of the left kidney (clipped) of 2K1C SED rats (0.27±0.01 g/100 g body weight, n=27) and 2K1C Ex rats (0.26±0.01 g/100 g body weight, n=45) was significantly lower than that of the left kidney of SHAM SED rats (0.33±0.01 g/100 g body weight, n=30; P<0.05). Also from week 2 to week 10, the relative wet weight of the right kidney (non-clipped) of 2K1C SED rats (0.41±0.02 g/100 g body weight, n=27) and 2K1C Ex rats (0.41±0.03 g/100 g body weight, n=45) was significantly higher than that of SHAM SED rats (0.35±0.02 g/100 g body weight, n=30; P<0.05). There was no difference between the right and left kidney of Sham Ex and SED rats. In weeks 6 and 10, the number of inflammatory cells in the left kidney was larger in 2K1C rats (SED and Ex) than that in SHAM SED rats (Figure 5). However, the Ex protocol significantly decreased the number of inflammatory cells in the right kidney of 2K1C rats compared with 2K1C SED rats in weeks 4 and 10 (P<0.05) (Figure 5). Collagen deposition in the left (clipped) kidney of 2K1C SED rats was significantly higher than that in SHAM SED rats from week 2 to week 10 (P<0.05). The Ex protocol significantly reduced collagen deposition in the left kidney of 2K1C rats compared with 2K1C SED rats in weeks 2, 4, and 10 (P<0.05). However, in week 10, collagen deposition in the left kidney of 2K1C Ex rats was similar to that of SHAM SED rats (P>0.05) (Figures 5B and 6). In the right kidney, the collagen deposition in 2K1C SED rats was also significantly higher than in SHAM SED rats from week 2 to week 10 (P<0.05). However, collagen deposition in 2K1C Ex rats was significantly lower than that in 2K1C SED rats from week 2 to week 10 (P<0.05) (Figures 5D and 7).

The MAP was positively correlated with the area of collagen deposition in the myocardium (r=0.9821), left kidney (r=0.9648), and right kidney (r=0.9597) for all animals at week 4 (P<0.05). We also observed an inverse correlation between the sensitivity of reflex bradycardia and the area of collagen deposition in the left kidney (r=-0.9988) and right kidney (r=-0.9807) for all animals at week 4 (P<0.05).

### Analysis of oxidative damage and formation of TBARS

**Figure 8:**
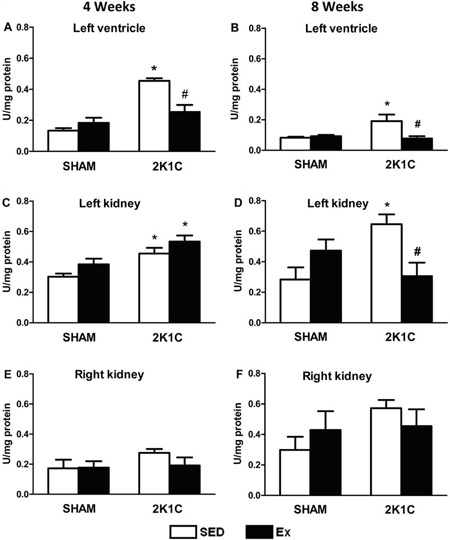
Relative level of TBARS, reported as malondialdehyde equivalents (U/mg protein), in the left ventricle (*A* and *B*), left kidney (*C* and *D*), and right kidney (*E* and *F*) of normotensive (SHAM, n=10) and hypertensive (2K1C, n=10) rats, either sedentary (SED) or subjected to 4 or 8 weeks of physical exercise training (Ex). *P<0.05 compared to SHAM SED. ^#^P<0.05 compared to 2K1C SED (ANOVA followed by Bonferroni test).

After 4 weeks of the Ex protocol, the level of TBARS, an indicator of lipid peroxidation, was significantly higher in the left ventricle and left kidney of 2K1C SED rats than SHAM SED rats (P<0.05). However, the level of TBARS in the left ventricle was significantly lower in 2K1C Ex than 2K1C rats (P<0.05) and similar between 2K1C Ex and SHAM SED rats (Figure 8A and C).

After 8 weeks of the Ex protocol, the level of TBARS in the left ventricle and left kidney was significantly higher in 2K1C SED than SHAM SED rats (P<0.05). However, the level of TBARS in the left ventricle and left kidney was significantly lower in 2K1C Ex than 2K1C SED rats (P<0.05) (Figure 8B and D). No difference in the level of TBARS was observed in the right and left kidneys in SHAM and 2K1C (SED and Ex) rats (P>0.05) (Figure 8E and F).

### CAT activity

**Figure 9:**
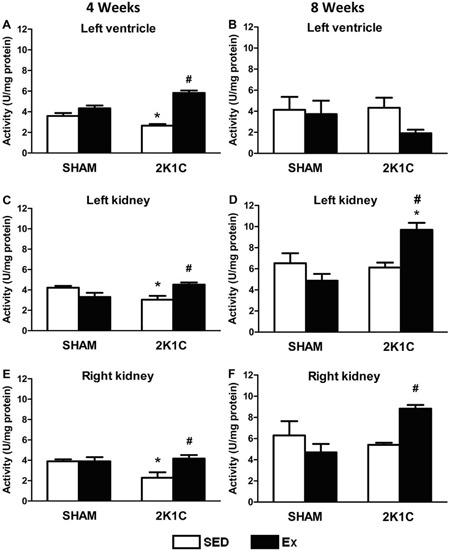
Catalase activity (U/mg protein) in the left ventricle (*A* and *B*), left kidney (*C* and *D*), and right kidney (*E* and *F*) of normotensive (SHAM, n=10) and hypertensive (2K1C, n=10) rats, either sedentary (SED) or subjected to 4 or 8 weeks of physical exercise training (Ex). *P<0.05 compared to SHAM SED group. ^#^P<0.05 compared to 2K1C SED (ANOVA followed by Bonferroni test).

After 4 weeks of the Ex protocol, the activity of CAT (an antioxidant enzyme) in the left ventricle, left kidney, and right kidney was significantly lower in 2K1C SED rats than in SHAM SED rats (P<0.05). However, the CAT activity in the left ventricle, left kidney, and right kidney was significantly higher in 2K1C Ex rats than in 2K1C SED rats (P<0.05) (Figure 9A, C and E).

After 8 weeks of the Ex protocol, the CAT activity in the left ventricle, left kidney, and right kidney was similar between the 2K1C SED and SHAM SED rats (Figure 9B, D and F). However, the CAT activity in the left kidney and right kidney was significantly higher in the 2K1C Ex than 2K1C SED rats (P<0.05) (Figure 9D and F).

## Discussion

In the present study, swimming without a workload effectively reduced MAP; restored reflex bradycardia and prevented concentric ventricular hypertrophy; restored the level of TBARS, an indicator of lipid peroxidation, in the left ventricle; and increased the activity of CAT, an antioxidant, in the left ventricle and both kidneys to levels similar to those in the SHAM SED rats for up to 4 weeks. Although Ex did not completely reverse MAP and reflex bradycardia at 8 weeks, it effectively decreased the TBARS level in the left ventricle and left kidney and increased the CAT activity in left and right kidneys. Ex also prevented collagen deposition in the myocardium and clipped kidney in 2K1C rats and maintained these levels similar to those observed in the SHAM SED rats up to week 10 of swimming.

The literature indicates the effectiveness of Ex in reducing MAP and improving baroreflex sensitivity in hypertensive humans (15) and animals (2,16,22,27). Previous studies (26,29) have demonstrated the important role of baroreceptors in the cardiovascular and autonomic adaptations induced by Ex. The sinoaortic denervation in spontaneously hypertensive rats subjected to treadmill running attenuated the adaptations induced by Ex, such as reduction of MAP and HR, reestablishment of baroreflex sensitivity, increased diameter of the left ventricular chamber, and reduced collagen deposition in the myocardium. However, other studies (7,16,18,20) have suggested that adjustment of the Ex intensity is necessary to maintain these benefits over time.

The results of the present study show that Ex effectively reduced the MAP and restored the reflex bradycardia up to week 4 of swimming, in agreement with previous studies from our laboratory that used the same model of hypertension and the same swimming protocol for 4 weeks (22) and 5 weeks (2). However, from week 6 to week 10, the basal MAP in the 2K1C Ex rats was similar to that in the 2K1C SED rats. Nevertheless, the reflex bradycardia of the 2K1C Ex rats was similar to that of the 2K1C SED and SHAM SED rats at week 6 and lower than that of the SHAM SED rats at weeks 8 and 10, suggesting that the adaptive benefits of Ex for improving reflex bradycardia until week 6 are compensated by hypertensive factors, especially by Ang II, which can modulate glutamatergic neurons in the rostral ventrolateral medulla and worsen the reflex bradycardia (30). A previous study from our laboratory (2) showed that Ex with a 0% or 3% workload for 5 weeks had beneficial effects on high blood pressure, high HR, and cardiac dysfunction in 2K1C rats. Nevertheless, Ex with a 0% workload more effectively improves the cardiac alterations observed in renovascular hypertensive rats; Ex with a 3% workload did not change the relatively lower sensitivity of the reflex bradycardia or prevent the cardiac lesions induced by hypertension. A possible explanation for these data is that Ex without workload until week 4 should induce adjustments of the activity of the sympathetic and parasympathetic nervous systems (7,17,29,31) and reduce oxidative stress (13), probably due to decreased levels of Ang II (11).

Concentric ventricular hypertrophy has been described under conditions of hypertension and myocardial infarction. (5,8), while eccentric ventricular hypertrophy has been described under physiological conditions such as aerobic exercise (2,6,7,9). Until week 10 in the present study, the relative heart weights were higher in 2K1C (SED and Ex) rats than in SHAM SED rats, and the number of myocardial inflammatory cells and cardiomyocyte diameter were higher in SHAM Ex rats than in SHAM SED rats. Additionally, at week 4, the W/L ratio in the left ventricle was lower in 2K1C Ex rats than in 2K1C SED rats and similar to that in SHAM SED rats, showing that Ex prevented concentric hypertrophy in 2K1C rats. Nevertheless, at week 6 only, the W/L ratio in the left ventricle was lower in SHAM Ex rats than in SHAM SED rats, suggesting eccentric hypertrophy. These data suggest that Ex (swimming) induced adaptation in the left ventricle in normotensive rats later than in hypertensive rats, probably because of the additional overload on the heart caused by both high blood pressure and the increased metabolic demand in response to Ex. Together, these data suggest that from week 4 onward, the volume, frequency, or intensity of Ex should be adjusted to maintain these benefits. Furthermore, Ex in the present study effectively reduced the TBARS level in the left ventricle at weeks 4 and 8, while the CAT activity was higher in 2K1C Ex than 2K1C SED rats only at week 4. The excessive production of TBARS and decreased CAT activity in the left ventricle and left (clipped) kidney in 2K1C SED rats was probably due to the increased levels of Ang II that develop in this model of hypertension. This could also explain the higher collagen deposition in the myocardium in 2K1C SED rats than in SHAM SED rats from week 2 to week 10. Moreover, 2K1C Ex rats showed a level of myocardial collagen deposition similar to that of SHAM SED rats from week 4 to week 10, indicating that these beneficial effects of Ex on the left ventricle were maintained during the entire 10 weeks, possibly by a decrease in oxidative stress. In fact, Ang II is a strong inductor of NADPH oxidase-induced reactive nitrogen and ROS generation in the plasma, heart, and kidney (32,33). Previously studies have demonstrated that high levels of Ang II-induced ROS are important for increased mRNA expression of collagen and fibronectin (34,35) and production of transforming growth factor-β1 (36). Conversely, the increase in oxygen consumption during Ex causes elevation of ROS, which is involved in the adaptive up-regulation of antioxidant gene expression (14).

Prieto et al. (37) showed that 3 weeks after clipping in 2K1C rats, the Ang I, Ang II, and ACE mRNA levels had increased and the Ang- (1-7) and ACE2 mRNA levels had decreased in the cortical and medullary regions of both the clipped and non-clipped kidneys. Additionally, the Ang II concentration was higher in the clipped than non-clipped kidney. Filho et al. (11) showed that Ex increased tissue Ang- (1-7) levels and decreased plasma Ang II levels in hypertensive rats. In agreement, our 2K1C SED rats showed greater deposition of collagen in both kidneys, clipped and non-clipped, from week 2 to week 10; however, Ex effectively reduced collagen deposition in the non-clipped kidneys of 2K1C rats from week 2 to week 10 and in the clipped kidney only at week 10 compared with 2K1C SED rats. The CAT activity in the left and right kidneys at weeks 4 and 8 was higher in 2K1C Ex rats than in 2K1C SED rats, while the TBARS level in the left kidney at week 8 was lower in 2K1C Ex rats than in 2K1C SED and similar to that in SHAM SED rats, suggesting a late beneficial effect of Ex over time, preventing collagen deposition likely due to the reduction of oxidative stress.

A limitation of the present study was the use of urethane anesthesia. Although urethane acts similarly on the sympathetic and parasympathetic nervous systems (38,39), the reduction of parasympathetic activity by this anesthetic could explain the unchanged HR in the SHAM Ex rats for each week. However, a lower HR was observed at 6, 8, and 10 weeks for both groups submitted to Ex (SHAM Ex and 2K1C Ex) than at week 2 of Ex. Additionally, we cannot rule out the possibility that the effects of anesthesia may differ between normotensive and hypertensive rats. Another limitation of this study was that reflex tachycardia was not evaluated.

In summary, our data indicate that Ex reduced the HR at 6, 8, and 10 weeks for both groups of rats submitted to Ex (SHAM Ex and 2K1C Ex) and that in 2K1C rats, Ex performed without a workload for 10 weeks provided distinct beneficial effects over time. Ex induced early effects on cardiovascular function by decreasing blood pressure and increasing reflex bradycardia and induced late effects by decreasing the oxidative stress and reducing the worsening of injuries that occur in the heart and kidneys during renovascular hypertension, thus preventing structural alterations in these organs.
